# DC algorithm for estimation of sparse Gaussian graphical models

**DOI:** 10.1371/journal.pone.0315740

**Published:** 2024-12-23

**Authors:** Tomokaze Shiratori, Yuichi Takano

**Affiliations:** 1 Graduate School of Science and Technology, University of Tsukuba, Tsukuba, Ibaraki, Japan; 2 Institute of Systems and Information Engineering, University of Tsukuba, Tsukuba, Ibaraki, Japan; Northwestern Polytechnical University, CHINA

## Abstract

Sparse estimation of a Gaussian graphical model (GGM) is an important technique for making relationships between observed variables more interpretable. Various methods have been proposed for sparse GGM estimation, including the graphical lasso that uses the *ℓ*_1_ norm regularization term, and other methods that use nonconvex regularization terms. Most of these methods approximate the *ℓ*_0_ (pseudo) norm by more tractable functions; however, to estimate more accurate solutions, it is preferable to directly use the *ℓ*_0_ norm for counting the number of nonzero elements. To this end, we focus on sparse estimation of GGM with the cardinality constraint based on the *ℓ*_0_ norm. Specifically, we convert the cardinality constraint into an equivalent constraint based on the largest-*K* norm, and reformulate the resultant constrained optimization problem into an unconstrained penalty form with a DC (difference of convex functions) representation. To solve this problem efficiently, we design a DC algorithm in which the graphical lasso algorithm is repeatedly executed to solve convex optimization subproblems. Experimental results using two synthetic datasets show that our method achieves results that are comparable to or better than conventional methods for sparse GGM estimation. Our method is particularly advantageous for selecting true edges when cross-validation is used to determine the number of edges. Moreover, our DC algorithm converges within a practical time frame compared to the graphical lasso.

## Introduction

### Background

Quantifying structural relationships between variables from observed data is a fundamental task in data mining. One commonly used measure is Pearson’s product-moment correlation coefficient, defined as the covariance of standardized variables. However, this measure has obvious limitations, such as its inability to deal with spurious correlations. In contrast, the Gaussian graphical model (GGM) involves learning partial correlations that correspond to elements of the precision matrix (i.e., the inverse of the covariance matrix). This approach provides a conditional independence graph, which graphically represents the relationships between variables while taking into account the influence of other variables. Such structural estimation has been effectively used in various fields, including analysis of brain activity patterns [1], anomaly detection [2], and sentiment analysis on social networks [3].

Since most variables usually have some relationship between them, the direct application of GGM often produces dense graphs, which have edges for many pairs of variables. For this reason, sparse estimation methods for GGM have been actively studied to estimate simple and essential relationships between variables [4–6]. The sparse estimation of GGM aims to create a conditional independence graph in a sparse form by reducing the number of nonzero elements in the estimated precision matrix. This approach allows us to estimate an interpretable graph even when the number of variables is larger than the sample size. However, sparse estimation of GGM faces several technical challenges, such as reducing the computational complexity and ensuring the positive definiteness of the precision matrix.

### Related work

Methods for estimating sparse precision matrices have long existed, including statistical testing methods [7] and threshold-based methods [8] for selecting nonzero elements. The lasso [9], a least-squares regression model with the *ℓ*_1_ norm regularization term, has also been used to estimate relationships between variables [10, 11]. However, these methods face computational challenges, such as the enormous computation time required for high-dimensional data and the inability to guarantee the positive definiteness of the precision matrix.

We focus on the method of adding regularization terms to the negative log-likelihood, which has become mainstream in recent years. Sparse GGM estimation was formulated as a convex optimization problem by adding the *ℓ*_1_ norm of elements of the precision matrix to the negative log-likelihood [12, 13]. The graphical lasso [14] is widely used to solve this optimization problem because it works quickly and stably even when the number of variables is larger than the sample size or when correlations between variables are high. It is also known that upon (asymptotic) convergence, the graphical lasso provides a positive definite precision matrix [15]. The graphical lasso is an iterative algorithm that minimizes the negative log-likelihood and the *ℓ*_1_ norm regularization term for GGM, where the strength of sparsity is adjusted by a regularization parameter. Various methods for sparse GGM estimation have been derived from the graphical lasso [15–17].

Methods for tuning regularization parameters include using information criteria, performing cross-validation, and analyzing the stability of the estimation results. Regularization parameters tuned using information criteria such as AIC and BIC work well for low-dimensional data, but tend to estimate graphs with high false positive rates for high-dimensional data [18]. The extended BIC is more effective at reproducing the true graph than the original BIC when the number of true edges is small [19, 20]. Cross-validation allows for more accurate selection of true edges than the use of information criteria, but suffers from high model variability [19]. Methods for analyzing the stability of the estimation results (e.g., by subsampling) have shown high accuracy in reproducing the true graph for high-dimensional data [18, 20]. Recently proposed methods include minimizing a network-characteristic-based function with respect to the regularization parameter [21], and assuming multivariate probability distributions other than the normal [22, 23].

While there are many successful methods based on the lasso for sparse estimation, it is well known that estimators with the *ℓ*_1_ norm regularization term are biased. A desirable property of estimators, known as the oracle property [24], has led to methods that compensate for the shortcomings of the lasso. Such methods include SCAD [25] and MCP [26], which use continuous nonconvex functions as regularization terms, and the adaptive lasso [27], which gives different regularization weights to each element of the precision matrix. SELO [28] was designed with a regularization term that closely approximates the *ℓ*_0_ norm, which represents the number of nonzero elements. A nonconvex regularization term was also proposed using an inverse trigonometric function that converges to the *ℓ*_0_ norm [29]. Although these approaches aim to approximate the *ℓ*_0_ norm by more tractable functions, it is more preferable to directly use the *ℓ*_0_ norm for counting the number of nonzero elements. A different approach is to solve the Lagrangian dual problem for estimating cardinality-constrained graphical models [30]. However, since the *ℓ*_0_ norm is a discontinuous nonconvex function, the associated sparse estimation is known to be NP-hard [31] and involves a positive duality gap. To the best of our knowledge, there is no sparse estimation method that directly uses the cardinality constraint based on the *ℓ*_0_ norm for GGM.

The DC (difference of convex functions) algorithm has been used to solve sparse optimization problems with the *ℓ*_0_ norm [32–34]. This method expresses a nonconvex objective function as the difference of two convex functions and repeatedly solves a convex optimization problem based on a linear approximation of the concave function to find a high-quality solution to the original nonconvex optimization problem [35, 36]. The DC algorithm have been applied to a variety of problem classes, including quadratic and bilevel optimization [37]. Phan et al. [38] designed a DC algorithm based on approximated DC representations for sparse estimation of the covariance matrix, whereas we focus on sparse estimation of the precision matrix based on the *ℓ*_0_ norm. Recently, Gotoh et al. [34] proposed new DC formulations and algorithms for sparse optimization problems, reporting favorable experimental results compared to the lasso. This DC optimization approach also allows us to estimate regularization parameter values that guarantee optimality for specific problems, avoiding the use of excessively large regularization parameter values.

### Our contribution

The main goal of this paper is to propose a high-performance algorithm for sparse GGM estimation with the *ℓ*_0_ norm. To this end, we apply the DC optimization framework proposed by Gotoh et al. [34] to sparse GGM estimation. Specifically, we first equivalently rewrite the cardinality constraint based on the *ℓ*_0_ norm by using the largest-*K* norm defined by Gotoh et al. [34]. We then reformulate this constrained optimization problem into an unconstrained penalty form with a DC representation, which is the difference of two convex functions. To solve this problem efficiently, we design a DC algorithm, which repeatedly executes the graphical lasso algorithm to solve convex optimization subproblems.

The effectiveness of our method is validated through computational experiments using two types of synthetic datasets. We investigate the results when the number of edges is determined by 5-fold cross-validation and when it is given in common to all methods. Experimental results show that our method can generate true graphs with accuracy comparable to or better than conventional methods for sparse GGM estimation. In particular, our method provides superior accuracy when estimating the number of edges through cross-validation. Furthermore, the computation time of our DC algorithm is only a few times longer than the graphical lasso, confirming that the algorithm converges within a practical time frame.

## Methods

In this section, we first give an overview of conventional models for sparse GGM estimation, then describe our method for sparse GGM estimation using the DC algorithm. Throughout this paper, we denote the set of consecutive integers as [*n*] ≔ {1, 2, …, *n*}.

### Sparse estimation of Gaussian graphical models

#### Gaussian graphical model

Let $x≔{\left(x_{1},x_{2},\ldots ,x_{p}\right)}^{⊤}\in R^{p}$ be a vector composed of *p* random variables that follow a multivariate normal distribution. A Gaussian graphical model (GGM) is a method for estimating a graph of the relationships between variables. Let $N(\mu ,\sigma^{2})$ denote a normal distribution with mean *μ* and variance *σ*^2^, and $\Omega ≔{\left(\omega_{jk}\right)}_{\left(j,k)\in [p]\times [p\right]}\in R^{p\times p}$ denote the precision matrix, which is the inverse of the covariance matrix $\Sigma ≔{\left(\sigma_{jk}\right)}_{\left(j,k)\in [p]\times [p\right]}\in R^{p\times p}$ of random vector ***x***. Then, the conditional distribution of *x*_*j*_ given the other variables ***x***_−*j*_ ≔ (*x*_*k*_)_*k*≠*j*_ can be written as follows:

$$\begin{array}{c} \text{Pr}\left(x_{j}|x_{-j}\right)=N(-\frac{1}{\omega_{jj}}\sum_{k\neq j}\omega_{jk}x_{k},\;\frac{1}{\omega_{jj}}). \end{array}$$ (1)

Note here that the relationship between *x*_*j*_ and *x*_*k*_ can be determined from the corresponding element *ω*_*jk*_ of the precision matrix.

Typically, the precision matrix is estimated through maximum likelihood estimation. Given *n* observed data points $x_{i}\in R^{p}\;\left(i\in \left[n\right]\right)$, the sample mean vector and the sample covariance matrix are defined as

$$m≔\frac{1}{n}\sum_{i=1}^{n}x_{i}\;\text{and}\;S≔\frac{1}{n}\sum_{i=1}^{n}\left(x_{i}-m\right){\left(x_{i}-m\right)}^{⊤},$$

respectively. Then, the log-likelihood function of the precision matrix **Ω** is written as

$$\begin{array}{cc} \ell (\Omega ) & ≔\text{log}(\prod_{i=1}^{n}{\left(2\pi \right)}^{-\frac{p}{2}}\text{det}{\left(\Omega \right)}^{\frac{1}{2}}\;\text{exp}\;[-\frac{1}{2}{\left(x_{i}-m\right)}^{⊤}\Omega \left(x_{i}-m\right)])\\ & =-\frac{pn}{2}\;\text{log}\left(2\pi \right)+\frac{n}{2}\;\text{log}\;\text{det}\left(\Omega \right)-\frac{1}{2}\sum_{i=1}^{n}{\left(x_{i}-m\right)}^{⊤}\Omega \left(x_{i}-m\right)\\ & =-\frac{pn}{2}\;\text{log}\left(2\pi \right)+\frac{n}{2}\;\text{log}\;\text{det}\left(\Omega \right)-\frac{n}{2}\text{tr}\left(\Omega S\right),\;∵x^{⊤}\Omega x=\text{tr}\left(\Omega xx^{⊤}\right) \end{array}$$

where det(⋅) and tr(⋅) are the determinant and the trace (i.e., the sum of diagonal elements) for a square matrix, respectively. By removing from the log-likelihood function the constant terms and coefficients that are irrelevant to the optimization and multiplying it by (−1), we obtain the following loss function (i.e., the negative log-likelihood) to be minimized:

$$\begin{array}{c} -\text{log}\;\text{det}(\Omega )+\text{tr}(\Omega S). \end{array}$$ (2)

After differentiation, we can derive the maximum likelihood estimator $\hat{\Omega }$ of the precision matrix as

$$-\Omega^{-1}+S=O\;\Rightarrow \;\hat{\Omega }=S^{-1},$$

where ***O*** is the zero matrix of appropriate size.

#### Regularization

If *ω*_*jk*_ = 0 (*j* ≠ *k*) in Eq (1), *x*_*k*_ does not influence *x*_*j*_ given ***x***_−*j*_, and this situation is called conditional independence. Therefore, a conditional independence graph, which connects only the variables that are not conditionally independent, is made sparse by assuming that *ω*_*jk*_ is exactly zero for many (*j*, *k*) ∈ [*p*] × [*p*]. To estimate such a sparse graph (or sparse precision matrix), we add a regularization term *p*_λ_(**Ω**) to the loss function (2) to penalize the absolute values of elements of the precision matrix as

$$\begin{array}{c} -\text{log}\;\text{det}\left(\Omega \right)+\text{tr}\left(\Omega S\right)+p_{\lambda }\left(\Omega \right), \end{array}$$ (3)

where λ > 0 is the regularization parameter for adjusting the strength of the penalty. As λ gets larger, more elements of **Ω** are estimated to be zero.

Various types of sparse estimators can be represented by the choice of the regularization term *p*_λ_(**Ω**). For example, the regularization term for the graphical lasso [14] is defined based on the *ℓ*_1_ norm as

$$\begin{array}{c} p_{\lambda }\left(\Omega \right)=\lambda {\left\|\text{vec}\left(\Omega \right)\right\|}_{1}, \end{array}$$ (4)

where the vec(⋅) operator rearranges the elements of a matrix into a vector as follows:

$$\text{vec}\left(\Omega \right)≔{\left(\omega_{11},\omega_{21},\ldots ,\omega_{pp}\right)}^{⊤}\in R^{n^{2}}.$$

Next, let us define for x∈R,

$$\begin{array}{cc} {\text{SCAD}}_{\lambda ,a}\left(x\right) & ≔\{\begin{array}{cc} \;\lambda |x| & \text{if}\;|x|\leq \lambda ,\\ \;\frac{a\lambda |x|-(x^{2}+\lambda^{2})/2}{a-1} & \text{if}\;\lambda <|x|\leq a\lambda ,\\ \;\frac{\left(a+1\right)\lambda^{2}}{2} & \text{if}\;|x|>a\lambda , \end{array} \end{array}$$

with a parameter *a* > 2. Then, the SCAD regularization term [25] is defined as

$$\begin{array}{c} p_{\lambda }\left(\Omega \right)=\sum_{j=1}^{p}\sum_{k=1}^{p}{\text{SCAD}}_{\lambda ,a}\left(\omega_{jk}\right). \end{array}$$ (5)

Additionally, let $\tilde{\Omega }≔{\left({\tilde{\omega }}_{jk}\right)}_{\left(j,k)\in [p]\times [p\right]}$ be a consistent estimator of **Ω**. Then, the regularization term for the adaptive lasso [27], a weighted version of lasso, is written as

$$\begin{array}{c} p_{\lambda }\left(\Omega \right)=\lambda \sum_{j=1}^{p}\sum_{k=1}^{p}\frac{1}{|{\tilde{\omega }}_{jk}{|}^{\gamma }}\left|\omega_{jk}\right|, \end{array}$$ (6)

with a parameter *γ* > 0.

**Fig 1:**
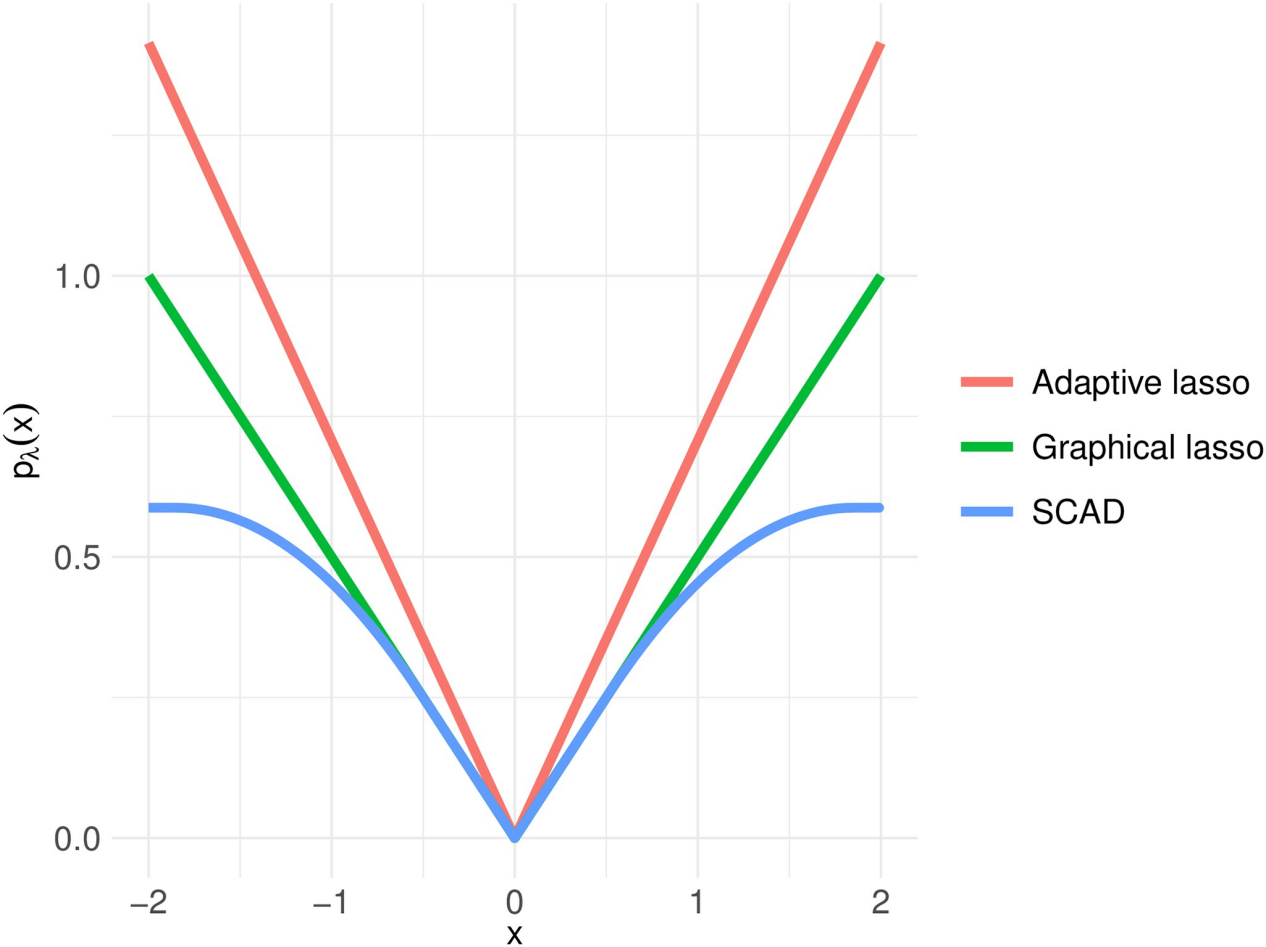
Graphs of the regularization terms.

Fig 1 illustrates graphs of *p*_λ_(*x*) of the graphical lasso, SCAD, and the adaptive lasso for *x* ∈ [−2, 2] with parameters λ = 0.5, *a* = 3.7, $\tilde{x}=0.5$, and *γ* = 0.5.

#### Graphical lasso

The graphical lasso [14], which is closely related to our algorithm, uses the regularization term (4) based on the *ℓ*_1_ norm. Let us define the sign function of x∈R as

$$\begin{array}{c} \text{sign}\left(x\right)≔\{\begin{array}{cc} \;1 & \text{if}\;x>0,\\ \;0 & \text{if}\;x=0,\\ \;-1 & \text{if}\;x<0. \end{array} \end{array}$$ (7)

Then, the following optimality condition is derived by differentiating Eq (3) with respect to **Ω** as

$$\begin{array}{c} -\Omega^{-1}+S+\lambda \Gamma \left(\Omega \right)=O, \end{array}$$ (8)

where

$$\begin{array}{c} \Gamma \left(\Omega \right)≔{\left(\gamma_{jk}\left(\omega_{jk}\right)\right)}_{\left(j,k)\in [p]\times [p\right]}\in R^{p\times p},\\ \gamma_{jk}\left(\omega_{jk}\right)\in \{\begin{array}{cc} \;\{\text{sign}\left(\omega_{jk}\right)\} & \text{if}\;\omega_{jk}\neq 0,\\ \;[-1,1] & \text{if}\;\omega_{jk}=0. \end{array} \end{array}$$ (9)

The graphical lasso simultaneously searches for solutions **Ω** and **Σ** = **Ω**^−1^ to the nonlinear Eq (8) by sequentially updating each column *j* ∈ [*p*] of the matrices. For this purpose, the matrices are decomposed into blocks (after row and column rearrangements) as

$$\begin{array}{c} \Omega =[\begin{array}{cc} \Omega_{-j} & \omega_{j}\\ \omega_{j}^{⊤} & \omega_{jj} \end{array}],\;\Sigma =[\begin{array}{cc} \Sigma_{-j} & \sigma_{j}\\ \sigma_{j}^{⊤} & \sigma_{jj} \end{array}], \end{array}$$ (10)

where $\Omega_{-j},\Sigma_{-j}\in R^{\left(p-1)\times (p-1\right)}$; $\omega_{j},\sigma_{j}\in R^{p-1}$; and $\omega_{jj},\sigma_{jj}\in R$. Then, the nonlinear Eq (8) with respect to the *j*-th column can be reduced to the lasso regression [9], and thus, each column can be computed efficiently using the coordinate descent method [14].

The procedure of the graphical lasso is summarized in Algorithm 1. The covariance matrix is initialized as **Σ**_0_ = ***S*** + λ***I***, which is derived from the diagonal elements determined from Eq (8) and the off-diagonal elements obtained by maximum likelihood estimation, where ***I*** is the identity matrix of appropriate size. The algorithm terminates when the update of the precision matrix becomes smaller than a threshold parameter *ε* > 0 in terms of the Frobenius norm ‖⋅‖_F_. Note also that since this algorithm has been criticized for the fact that the objective function does not decrease monotonically, several methods have been proposed to accelerate the convergence [15].

**Algorithm 1** Graphical Lasso for Sparse GGM Estimation

**Input:** Sample covariance matrix ***S***, regularization parameter λ > 0, convergence threshold *ε* > 0.

**Output:** Precision matrix **Ω**.

**Initialize:** Iteration number *t* ← 0, covariance matrix **Σ**_0_ = ***S*** + λ***I***, precision matrix $\Omega_{0}=\Sigma_{0}^{-1}$.

1: (**Ω**, **Σ**) ← (**Ω**_0_, **Σ**_0_).

2: **repeat**

3:  **for** *j* ∈ [*p*] **do**

4:   Decompose **Ω** and **Σ** into block matrices (after rearrangement) as in Eq (10).

5:   Update ***ω***_*j*_, *ω*_*jj*_, ***σ***_*j*_, *σ*_*jj*_ using the lasso regression [14].

6:   Rearrange the elements of **Ω** and **Σ** back into the original matrices.

7:  **end for**

8:  (**Ω**_*t*+1_, **Σ**_*t*+1_) = (**Ω**, **Σ**).

9:  *t* ← *t* + 1.

10: **until** $\|\Omega_{t}-\Omega_{t-1}{\|}_{F}^{2}<\varepsilon$.

11: **return** **Ω**_*t*_.

### DC algorithm for sparse GGM estimation

#### Formulation

For $w≔{\left(w_{i}\right)}_{i\in [m]}\in R^{m}$, we denote the *ℓ*_0_ (pseudo) norm by

$${\left\|w\right\|}_{0}≔\left|\left\{i\in \left[m\right]|w_{i}\neq 0\right\}\right|,$$

which counts the number of nonzero elements of ***w***. To find a positive definite precision matrix **Ω** ≻ ***O***, we impose the constraint **Ω** ⪰ *δ* ***I*** (i.e., **Ω** − *δ**I*** is positive semidefinite) with a small positve constant *δ* > 0. Then, sparse GGM estimation can be naturally posed as the following cardinality-constrained optimization problem:

$$\begin{array}{cc} \underset{\Omega ⪰\delta I}{\text{minimize}}\; & -\text{log}\;\text{det}(\Omega )+\text{tr}(\Omega S) \end{array}$$ (11)

$$\begin{array}{cc} \text{subject}\;\text{to}\; & {\left\|\text{vec}\left(\Omega \right)\right\|}_{0}\leq K, \end{array}$$ (12)

where *K* ∈ [*p*^2^] is a cardinality parameter for limiting the number of nonzero elements of the precision matrix.

Following Gotoh et al. [34], we now define the largest-*K* norm as follows.

**Definition 1**. For $w≔{\left(w_{i}\right)}_{i\in [m]}\in R^{m}$, let *π* be a permutation of [*m*] satisfying |*w*_*π*(1)_| ≥ |*w*_*π*(2)_| ≥ ⋯ ≥ |*w*_*π*(*m*)_|. Then, the largest-*K* norm is defined as the sum of the *K* largest absolute values as

$$\begin{array}{c} {\left||\right|w\left||\right|}_{K}≔\sum_{i=1}^{K}\left|w_{\pi (i)}\right|. \end{array}$$ (13)

Note here that

$${\left\|w\right\|}_{0}\leq K\Leftrightarrow \sum_{i=K+1}^{m}|w_{\pi (i)}{\left|=0\Leftrightarrow \|w\right\|}_{1}-{\left||\right|w\left||\right|}_{K}=0.$$

Therefore, problem (11) and (12) can be equivalently rewritten as

$$\begin{array}{cc} \underset{\Omega ⪰\delta I}{\text{minimize}}\; & -\text{log}\;\text{det}(\Omega )+\text{tr}(\Omega S) \end{array}$$ (14)

$$\begin{array}{cc} \text{subject}\;\text{to}\; & {\left\|\text{vec}\left(\Omega \right)\right\|}_{1}-{\left||\right|\text{vec}\left(\Omega \right)\left||\right|}_{K}=0. \end{array}$$ (15)

Although the *ℓ*_0_ norm in Eq (12) is a discontinuous function, Eq (15) is represented by the difference of two convex continuous functions and defines the same feasible region as the original problem (11) and (12).

In what follows, we focus on the following penalized version of problem (14) and (15):

$$\begin{array}{c} \underset{\Omega ⪰\delta I}{\text{minimize}}\;-\text{log}\;\text{det}\left(\Omega \right)+\text{tr}\left(\Omega S\right)+\eta ({\left\|\text{vec}\left(\Omega \right)\right\|}_{1}-{\left||\right|\text{vec}\left(\Omega \right)\left||\right|}_{K}), \end{array}$$ (16)

or equivalently,

$$\begin{array}{c} \underset{\Omega ⪰\delta I}{\text{minimize}}\;(-\text{log}\;\text{det}\left(\Omega \right)+\text{tr}\left(\Omega S\right)+{\eta \|\text{vec}\left(\Omega \right)\|}_{1})-\eta {\left||\right|\text{vec}\left(\Omega \right)\left||\right|}_{K}, \end{array}$$ (17)

where *η* > 0 is a penalty parameter. Problem (17) is called a DC optimization problem [35] because its objective is the difference of two convex functions.

#### Algorithm

Each iteration of the DC algorithm constructs a linear approximation of the concave function and solves the resultant convex optimization problem to update the solution.

Following Gotoh et al. [34], we calculate a subgradient of the largest-*K* norm based on the sign function (7) as

$$\begin{array}{c} s\left(w\right)≔{\left(s_{i}\left(w\right)\right)}_{i\in [m]}\in \partial {\left||\right|w\left||\right|}_{K}, \end{array}$$ (18)

where

$$\begin{array}{c} s_{i}\left(w\right)≔\{\begin{array}{cc} \;\text{sign}(w_{i}) & \text{if}\;\pi^{-1}\left(i\right)\in \left[K\right],\\ \;0 & \text{otherwise} \end{array}\;\left(i\in \left[m\right]\right). \end{array}$$ (19)

Let **Ω**_*t*_ be an incumbent solution at the *t*-th iteration of the DC algorithm. By introducing a linear approximation of the largest-*K* norm, a surrogate objective function is given by

$$\begin{array}{c} g_{t}\left(\Omega \right)≔-\text{log}\;\text{det}\left(\Omega \right)+\text{tr}\left(\Omega S\right)+\eta {\left\|\text{vec}\left(\Omega \right)\right\|}_{1}-\eta s{\left(\text{vec}\left(\Omega_{t}\right)\right)}^{⊤}\text{vec}\left(\Omega \right). \end{array}$$ (20)

By differentiating *g*_*t*_(**Ω**), we obtain the following optimalitiy condition based on Eq (9):

$$\begin{array}{c} \frac{\partial g_{t}}{\partial \Omega }=-\Omega^{-1}+\left(S-\eta V\left(\Omega_{t}\right)\right)+\eta \Gamma \left(\Omega \right)=O, \end{array}$$ (21)

where $V\left(\Omega_{t}\right)≔{\text{vec}}^{-1}\left(s\left(\text{vec}\left(\Omega_{t}\right)\right)\right)\in R^{p\times p}$. Note that this nonlinear equation corresponds to Eq (8), where ***S*** is replaced by ***S*** − *η**V***(**Ω**_*t*_). Accordingly, the graphical lasso algorithm can be applied to Eq (21) and gives a solution **Ω**, which is positive definite upon (asymptotic) convergence.

Our DC algorithm for estimating a sparse precision matrix is described in Algorithm 2. Although the graphical lasso assumes that the sample covariance matrix is positive definite (i.e., ***S*** ≻ ***O***), the corresponding matrix ***S*** − *η**V***(**Ω**_*t*_) in Eq (21) may not be positive definite depending on the value of the penalty parameter *η*. Note here that if *η* ≈ 0, then ***S*** − *η**V***(**Ω**_*t*_) ≈ ***S*** ≻ ***O***. In addition, all diagonal elements of ***V***(**Ω**_*t*_) are equal to 1 due to the positive definiteness of the precision matrix; therefore, if *η* > λ_min_(***S***), then ***S*** − *η**V***(**Ω**_*t*_) ⊁ ***O***, where λ_min_(⋅) denotes the smallest eigenvalue of a matrix. For this reason, our algorithm adaptively searches for the largest possible *η* ∈ [0, λ_min_(***S***)] such that ***S*** − *η**V***(**Ω**_*t*_) ≻ ***O***.

**Algorithm 2** DC Algorithm for Sparse GGM Estimation

**Input:** Sample covariance matrix ***S***, cardinality parameter *K* ∈ [*p*^2^], convergence threshold *ε* > 0, shrinking parameter *α* ∈ (0, 1).

**Output:** Precision matrix **Ω**.

**Initialize:** Iteration number *t* ← 0, precision matrix **Ω**_0_ ≻ ***O***.

1: **repeat**

2:  Compute the subgradient ***s***(vec(**Ω**_*t*_)) ∈ *∂*|||vec(**Ω**_*t*_)|||_*K*_ as in Eqs (18) and (19).

3:  *η* ← λ_min_(***S***).

4:  **repeat**

5:   *η* ← *αη*.

6:  **until *S*** − *η**V***(**Ω**_*t*_) ≻ ***O***.

7:  Solve Eq (21) using Algorithm 1 to compute **Ω**_*t*+1_.

8:  *t* ← *t* + 1.

9: **until** $\|\Omega_{t}-\Omega_{t-1}{\|}_{\text{F}}^{2}<\varepsilon$.

10: **return Ω**_*t*_.

## Experimental results and discussion

In this section, we report experimental results on two types of synthetic datasets to validate the effectiveness of our method for sparse GGM estimation (The source code of the experiments is available at https://github.com/torikaze/DC-GGM).

### Synthetic datasets

Following Mazumder and Hastie [15], and Yuan and Lin [13], we prepared two types of synthetic datasets based on random and chain graphs. For each dataset, we begin by defining a ground-truth precision matrix as follows.

**Random graph:** Create a symmetric matrix $A_{2}≔\left(A_{1}+A_{1}^{⊤}\right)/2\in R^{p\times p}$, where each element of $A_{1}\in R^{p\times p}$ is independently generated from the standard normal distribution. Randomly set some of the off-diagonal elements of ***A***_2_ to zeros while maintaining symmetry of the matrix. Define **Ω**_rnd_ ≔ ***A***_2_ + *η*_rnd_***I***, with *η*_rnd_ being set such that λ_min_(**Ω**_rnd_) = 1.
**Chain graph:** Set up a tridiagonal matrix as follows:
  $$\omega_{jk}≔\{\begin{array}{cc} \;1 & \text{if}\;j=k,\\ \;0.5 & \text{if}\;|j-k|=1,\\ \;0.25 & \text{if}\;|j-k|=2,\\ \;0 & \text{otherwise} \end{array}\;\left(\left(j,k\right)\in \left[p\right]\times \left[p\right]\right).$$
  Randomly set some of the nonzero off-diagonal elements to zeros while maintaining symmetry of the matrix to obtain a precision matrix **Ω**_chn_ ≔ (*ω*_*jk*_)_(*j*,*k*)∈[*p*]×[*p*]_.

**Fig 2:**
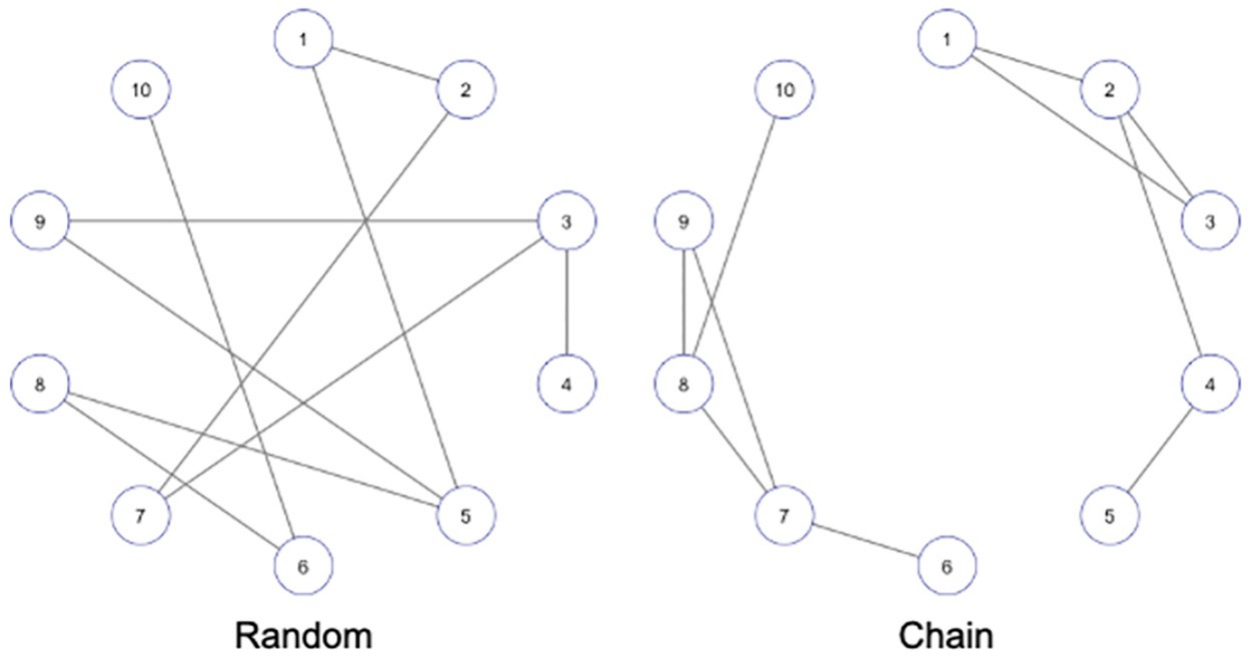
Examples of ground-truth graph structures with (*p*, *n*_≠0_) = (10, 10).

Fig 2 shows examples of graph structures based on the precision matrices **Ω**_rnd_ and **Ω**_chn_. Let *n*_≠0_ be the number of true edges (i.e., half the number of nonzero off-diagonal elements of the precision matrix). The procedure for creating synthetic datasets is described as follows:

1. Generate a ground-truth precision matrix $\Omega^{⋆}≔{\left(\omega_{jk}^{⋆}\right)}_{\left(j,k)\in [p]\times [p\right]}\in \left\{\Omega_{\text{rnd}},\Omega_{\text{chn}}\right\}$ with 2 ⋅ *n*_≠0_ nonzero off-diagonal elements, and create the corresponding covariance matrix as **Σ**^⋆^ ≔ (**Ω**^⋆^)^−1^.
2. Generate $x_{i}\in R^{p}\;\left(i\in \left[n\right]\right)$ independently from a multivariate normal distribution $N(0,\Sigma^{⋆})$, and compute the sample covariance matrix ***S***.
3. Compute ***S*** ← *ζ**D***_***S***_ + (1 − *ζ*)***S*** based on the shrinkage estimation [39], where ***D***_***S***_ is the diagonalized matrix of ***S***, and *ζ* ∈ [0, 1] is a shrinkage parameter.

For generation of synthetic datasets, we set the number of variables, the sample size, and the number of true edges as follows:

$$p\in \left\{50,100,200,400\right\},\;n\in \left\{p/2,p,2p\right\},\;\text{and}\;n_{\neq 0}=30.$$

Due to the randomness of dataset generation, we created 30 precision matrices for each case and show average results with 95% confidence intervals.

### Experimental setup

To validate the effectiveness of our method, we compared the estimation accuracy and characteristics of the following methods for sparse GGM estimation:

**DC:** Our DC algorithm (Algorithm 2);
**glasso:** Graphical lasso (Algorithm 1) [14];
**SCAD:** SCAD regularized estimation [25];
**adapt:** Adaptive lasso [27].

All experiments were conducted using the R programming language. We used the glasso package [14] to implement the graphical lasso, and the GGMncv package [40] to implement the SCAD regularized estimation and the adaptive lasso. In the DC algorithm, we set *α* = 0.5 as the shrinking parameter, and **Ω**_0_ = (***S*** + ***I***)^−1^ as the initial solution. Following Fan and Li [24], we set *a* = 3.7 in Eq (5) for the SCAD regularized estimation. In Eq (6) for the adaptive lasso, we set *γ* = 0.5 by following Fan et al. [25], and $\tilde{\Omega }=S^{-1}$ according to default configuration of the GGMncv package. We set *ε* = 10^−4^ as the convergence threshold.

To evaluate the accuracy of the estimated precision matrix $\hat{\Omega }≔{\left({\hat{\omega }}_{jk}\right)}_{\left(j,k)\in [p]\times [p\right]}\in R^{p\times p}$, we first define the true positive (TP), false positive (FP), and false negative (FN) rates as

$$\begin{array}{c} \text{TP}≔\sum_{j=1}^{p}\sum_{k=j+1}^{p}I({\hat{\omega }}_{jk}\neq 0\;\text{and}\;\omega_{jk}^{⋆}\neq 0),\\ \text{FP}≔\sum_{j=1}^{p}\sum_{k=j+1}^{p}I({\hat{\omega }}_{jk}\neq 0\;\text{and}\;\omega_{jk}^{⋆}=0),\\ \text{FN}≔\sum_{j=1}^{p}\sum_{k=j+1}^{p}I({\hat{\omega }}_{jk}=0\;\text{and}\;\omega_{jk}^{⋆}\neq 0), \end{array}$$

where I(*Q*) is an indicator function that returns 1 if the proposition *Q* is true, and 0 otherwise. The F1 score is then defined as

$$\begin{array}{c} \text{F1}\;\text{score}≔\frac{2\times \text{Recall}\times \text{Precision}}{\text{Recall}+\text{Precision}}, \end{array}$$

where

$$\text{Recall}=\frac{\text{TP}}{\text{TP}+\text{FN}},\;\text{Precision}=\frac{\text{TP}}{\text{TP}+\text{FP}}.$$

The F1 score is an appropriate evaluation metric for imbalanced datasets such as those used in our experiments. The F1 score was also used for evaluation of regularized graphical models [18] and subset selection for linear regression [41].

### Results with number of edges determined by cross-validation

We will now investigate the results where the number of edges in an estimated graph was determined through 5-fold cross-validation of the loss function (2). Here, the cardinality parameter *K* for the DC algorithm was chosen from 100 equally spaced values between *p* + 2 and *p*^2^. The regularization parameter λ for the other methods was chosen from 100 equally spaced values in the range [0, λ_max_], where λ_max_ was set such that the number of selected edges was zero.

**Fig 3:**
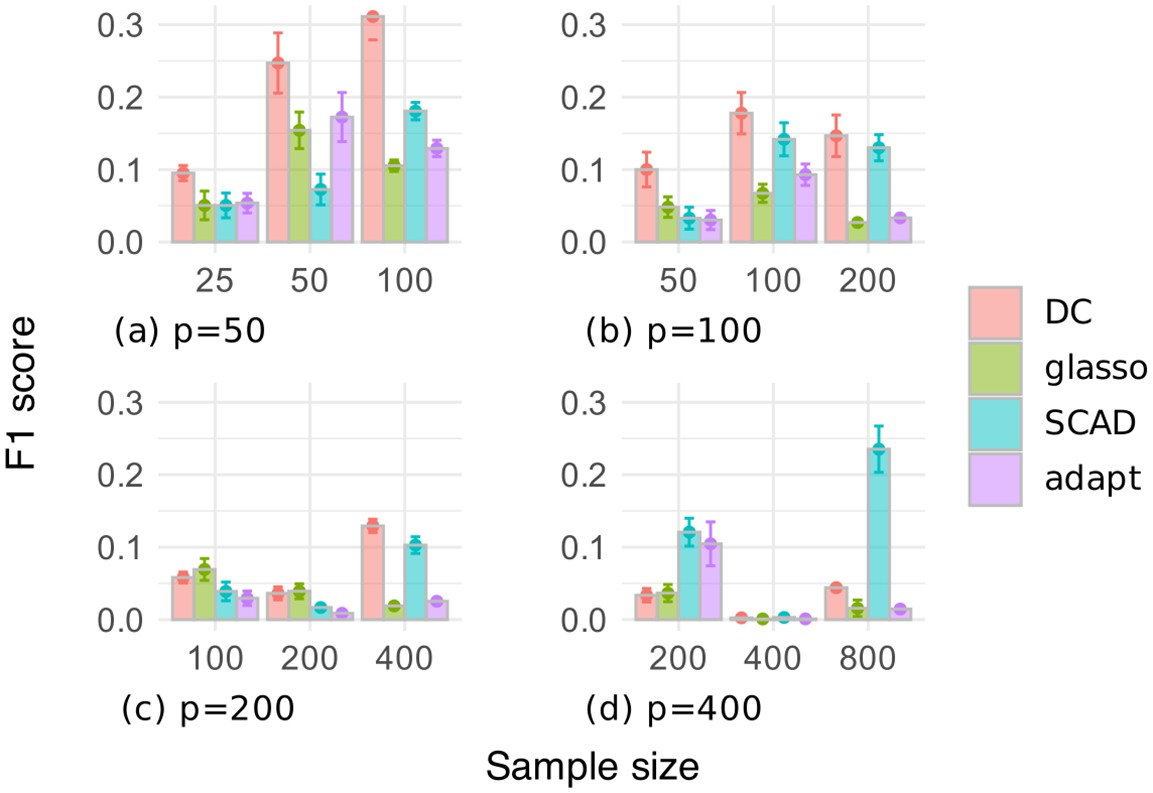
F1 score of edges selected through cross-validation on the random graph dataset.

**Fig 4:**
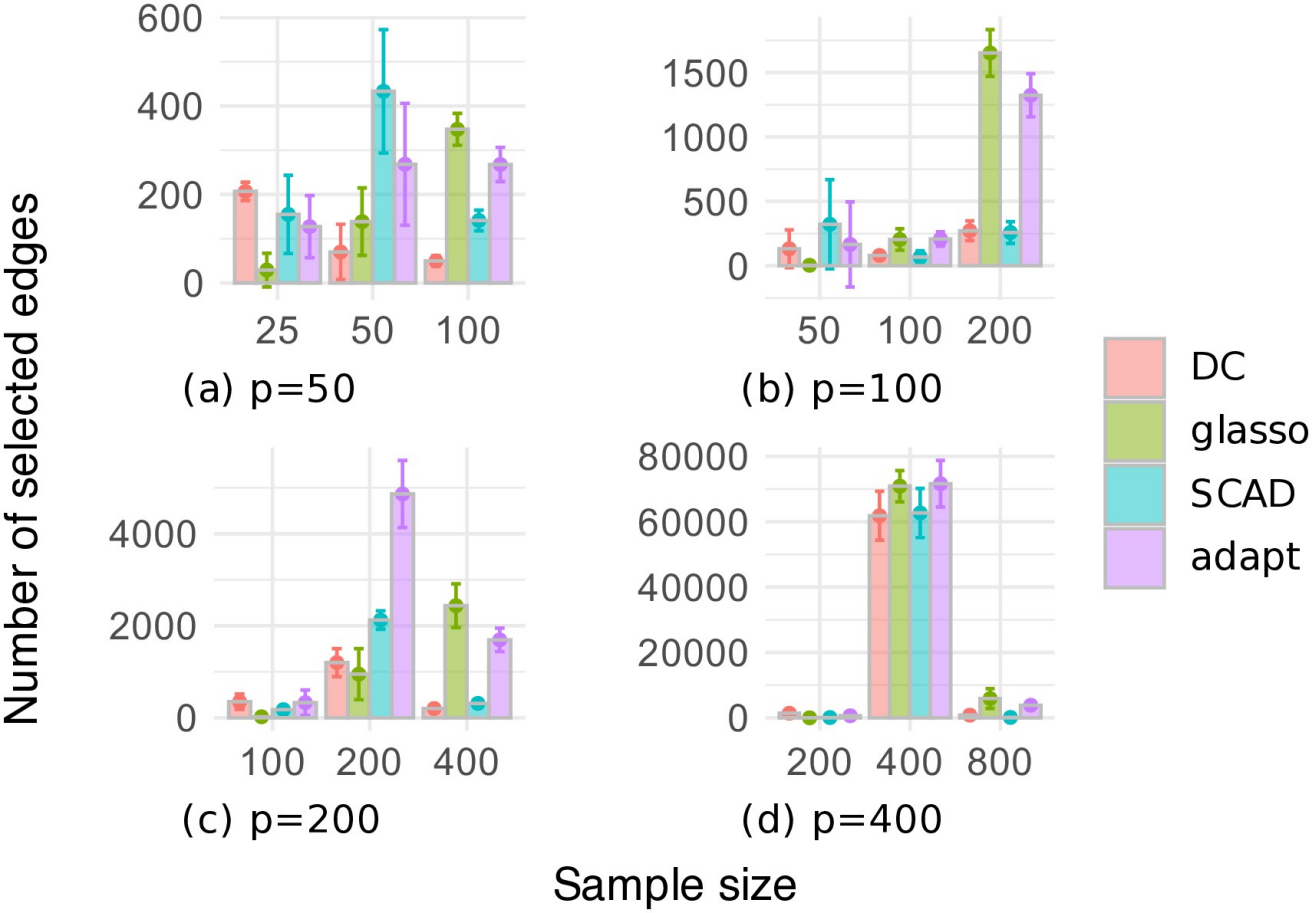
Number of edges selected through cross-validation on the random graph dataset.

Figs 3 and 4 respectively show the F1 scores and the numbers of selected edges for the random graph dataset, where the number of variables is *p* ∈ {50, 100, 200, 400}, and the sample size is *n* ∈ {*p*/2, *p*, 2*p*}. In Fig 3, our DC method often outperformed the other methods in terms of the F1 score, except when *p* = 400. Additionally, the estimation accuracy of our DC method tended to improve as the sample size increased. Fig 4 shows that the glasso, SCAD, and adapt methods often selected too many edges, resulting in low F1 scores. In contrast, our DC method showed relatively small variations in the number of selected edges, indicating that it is possible for our DC algorithm to produce estimates that are robust to changes in the data.

**Fig 5:**
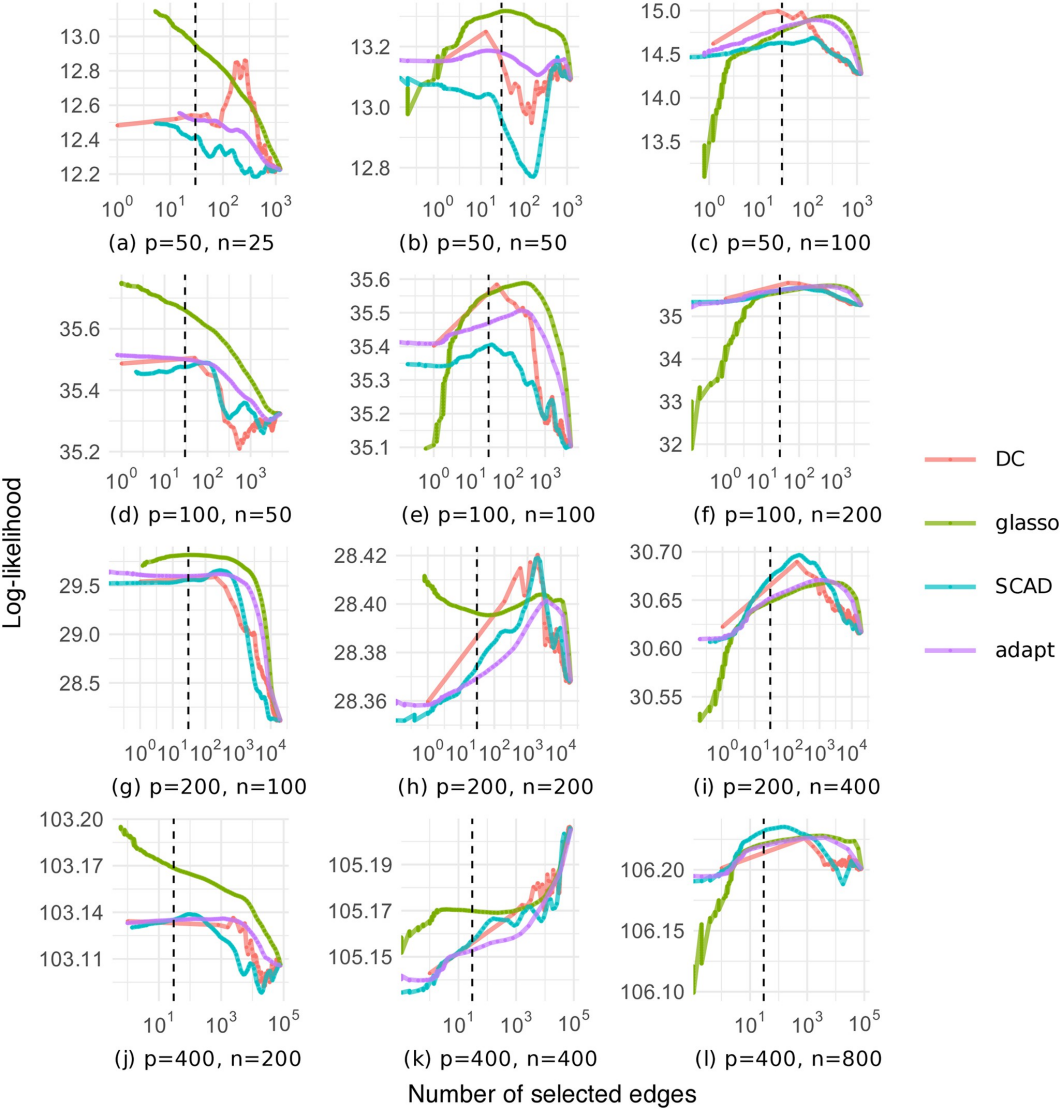
Log-likelihood as a function of the number of selected edges on the random graph dataset (black dashed line: The true number of edges).

To examine the number of edges selected through cross-validation in more detail, Fig 5 shows the relationship between the average number of selected edges and the average log-likelihood in cross-validation on the random graph dataset. Note that this figure shows the result of one of 30 trials, and that each method selected the number of edges that maximizes the log-likelihood. As a general trend, fewer edges were selected when *p* > *n*, whereas more edges were selected when *p* < *n*. Our DC method often maximized the log-likelihood at close to the true number of edges compared to the other methods. However, with our DC method, the relationship between the number of selected edges and the log-likelihood was not as smooth as with the other methods.

**Fig 6:**
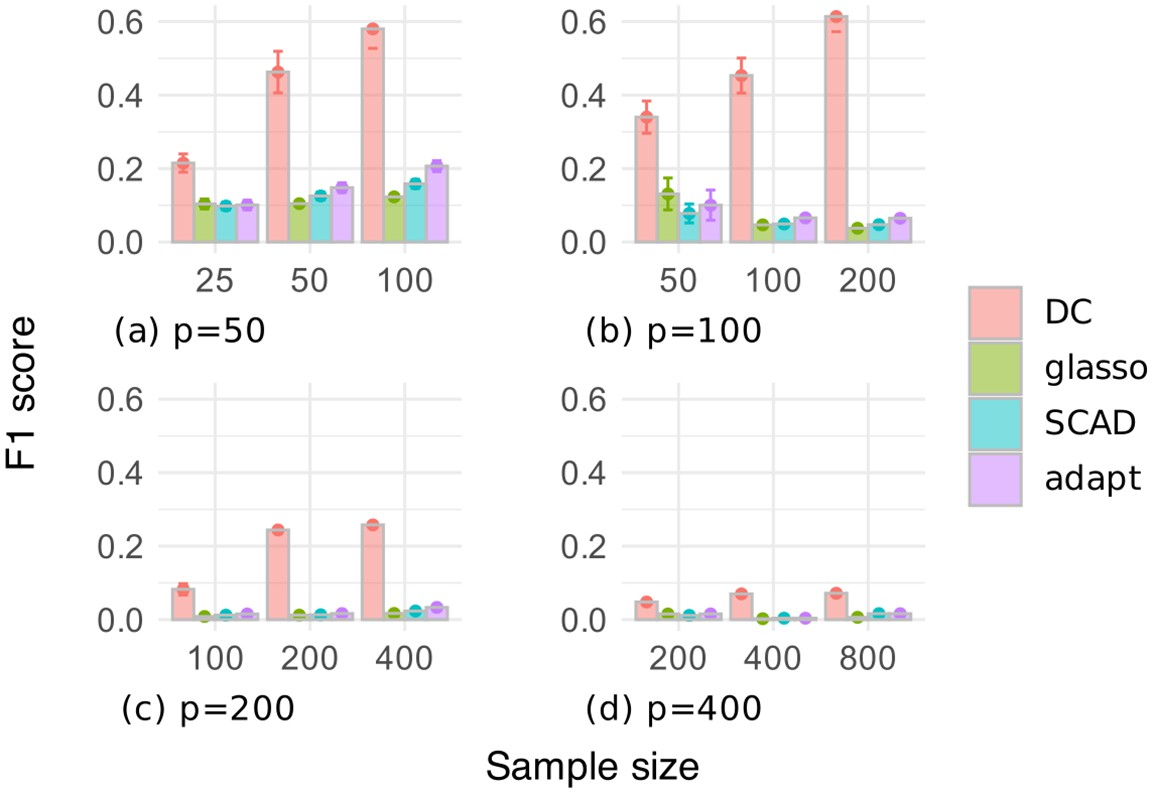
F1 score of edges selected through cross-validation on the chain graph dataset.

**Fig 7:**
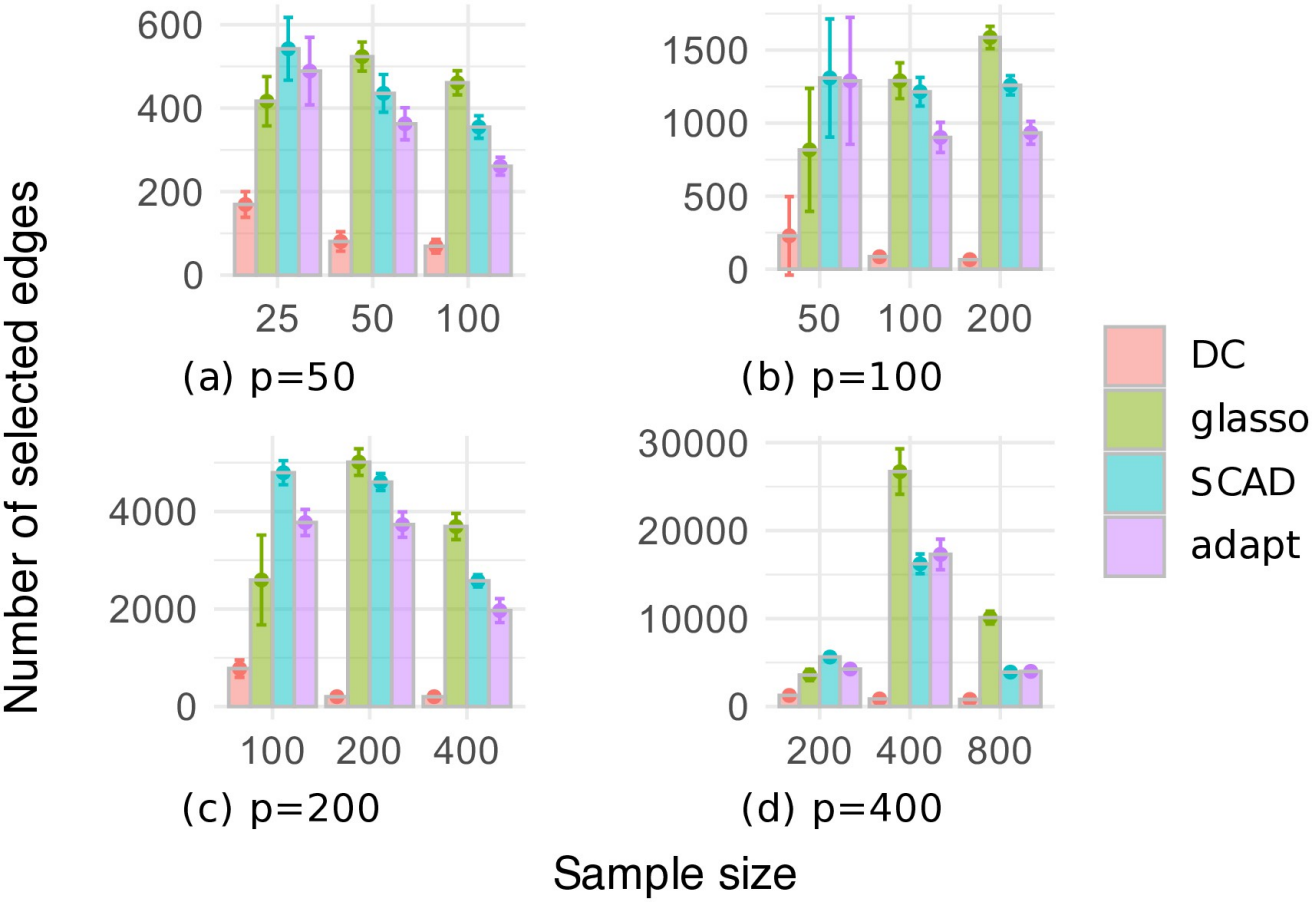
Number of edges selected through cross-validation on the chain graph dataset.

Figs 6 and 7 respectively show the F1 scores and the numbers of selected edges for the chain graph dataset. In Fig 6, our DC method significantly outperformed the other methods in terms of the F1 score. Fig 7 implies that the glasso, SCAD, and adapt methods had low F1 scores because they produced very dense graphs. In contrast, our DC method selected a relatively small and stable number of edges, consistent with the trends observed in the random graph dataset.

**Fig 8:**
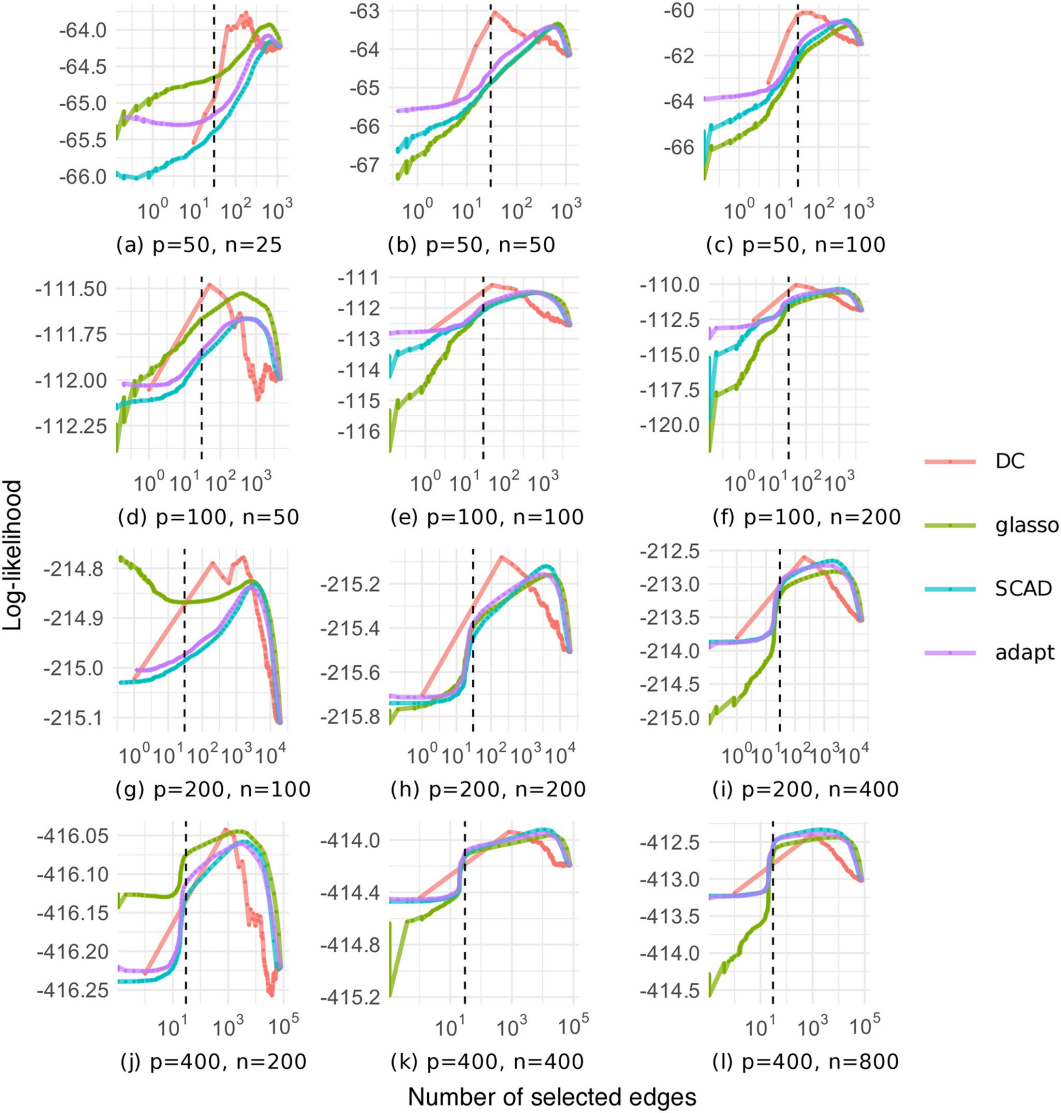
Log-likelihood as a function of the number of selected edges on the chain graph dataset (black dashed line: The true number of edges).

Fig 8 shows the relationship between the average number of selected edges and the average log-likelihood in cross-validation on the chain graph dataset. Our DC method often maximized the log-likelihood at close to the true number of edges compared to the other methods; however, as with the random graph dataset, the relationship between the number of selected edges and the log-likelihood was not very smooth, and the number of selected edges was biased relative to the true number of edges.

These results confirm that our method was very accurate in edge selection when cross-validation was used to determine the number of edges. In contrast, other methods often selected an excessively large number of edges, resulting in low F1 scores.

### Results with a given number of edges

We will now investigate the results where the number of edges in an estimated graph was given as 20, 30, and 40 commonly for all methods.

**Fig 9:**
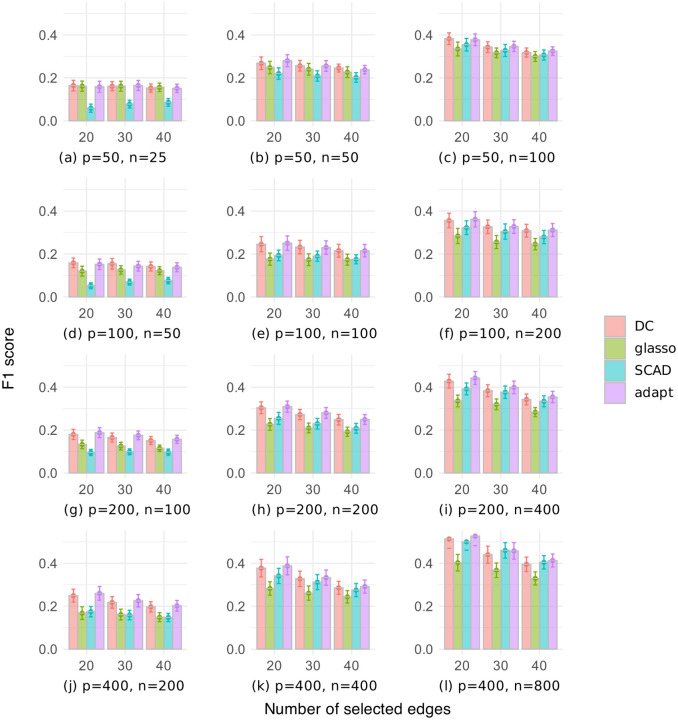
F1 score of a given number of selected edges on the random graph dataset.

Fig 9 shows the F1 scores with different numbers of selected edges for the random graph dataset, where the number of variables is *p* ∈ {50, 100, 200, 400}, and the sample size is *n* ∈ {*p*/2, *p*, 2*p*}. Overall, the F1 scores were better for Fig 9 than for Fig 3, with the DC and adapt methods performing particularly well in Fig 9. Conversely, the glasso and SCAD methods generally had low F1 scores. As the sample size increased, the F1 scores of all methods improved, possibly due to more accurate estimation of the sample covariance matrix. Additionally, as the number of selected edges increased, the F1 scores of all methods tended to decrease, likely due to an increase in the number of false positive edges.

**Fig 10:**
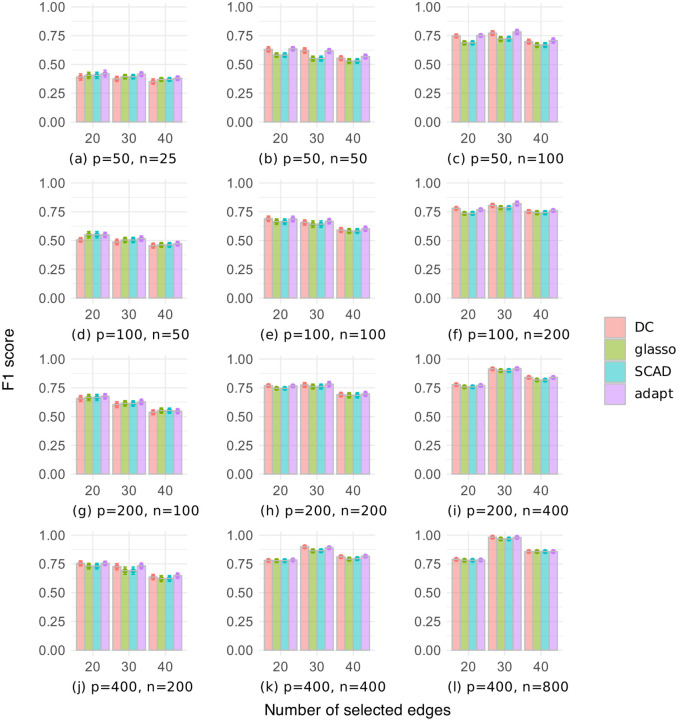
F1 score of a given number of selected edges on the chain graph dataset.

Fig 10 shows the F1 scores with different numbers of selected edges for the chain graph dataset. The F1 scores were generally high compared to the random graph dataset, with the DC and adapt methods showing slight superiority. Although the F1 scores of our DC method were comparable to or lower than those of the other methods when *p* > *n*, our DC method performed relatively well when *p* ≤ *n*. As with the random graph dataset, when *p* ≥ *n*, increasing the number of selected edges tended to decrease the F1 score. When *p* < *n*, setting the number of edges to 30, which is equal to the number of true edges, often yielded the best results. These results show that it was easier to select true edges in the chain graph dataset than in the random graph dataset, and that setting the number of edges to the true number resulted in fewer false positive and false negative edges when the sample size was large enough.

These results confirm that for the random graph dataset, the DC and adapt methods performed better than the other methods when selecting a given number of edges. On the other hand, for the chain graph dataset, all methods showed very high scores, with small differences.

### Computation time

We will now investigate the computation time required by our DC algorithm for estimating sparse precision matrices. Here, the cardinality parameter *K* in our DC method was set to half the total number of edges (i.e., *K* = *p*(*p* − 1)/4), and the regularization parameter λ in the glasso method was set to the median of the absolute values of off-diagonal elements of the sample covariance matrix. Since there were minor differences among the glasso, SCAD and adapt methods, only the results for the glasso method are shown.

**Fig 11:**
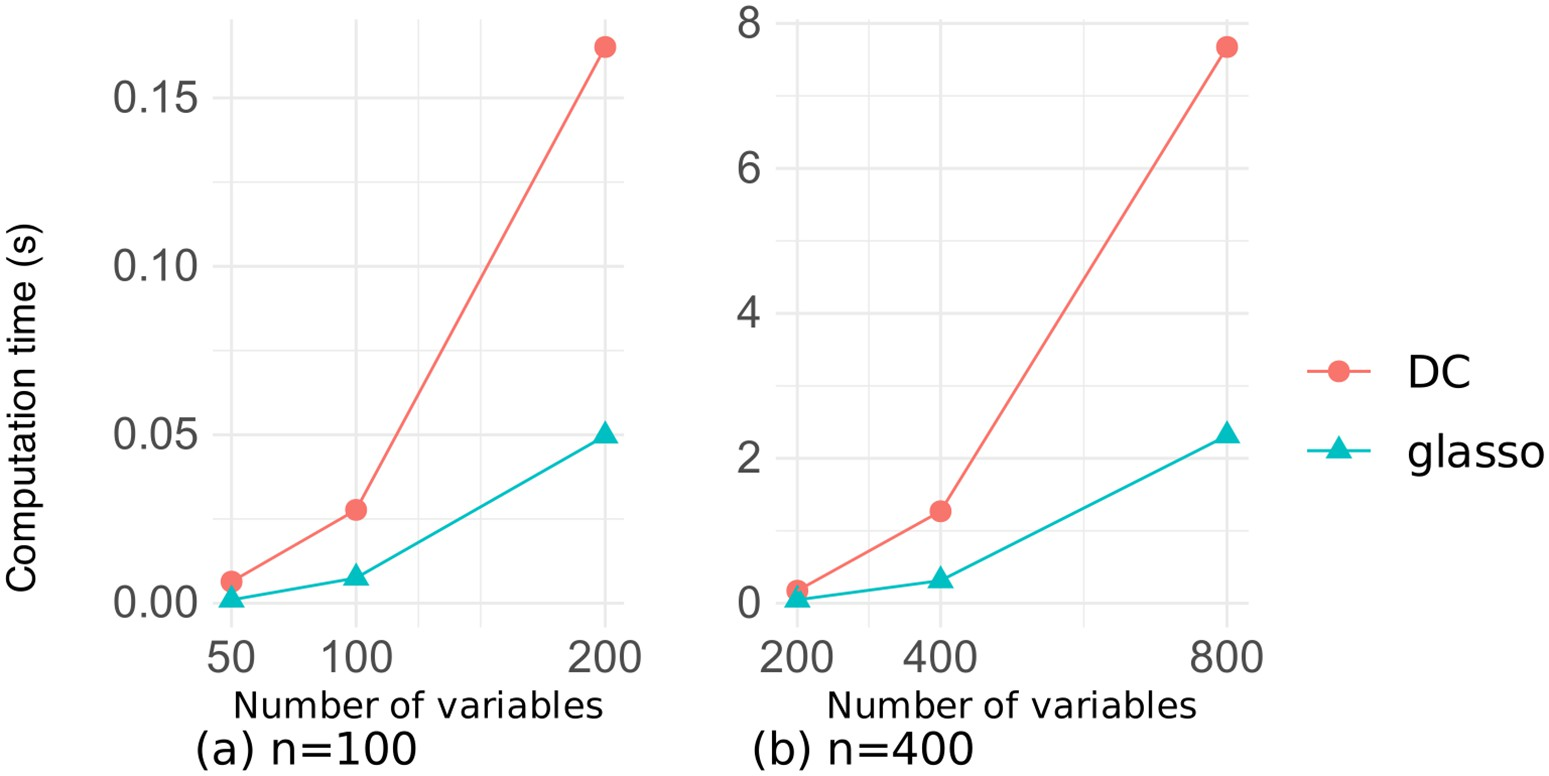
Computation time as a function of the number of variables on the random graph dataset.

**Fig 12:**
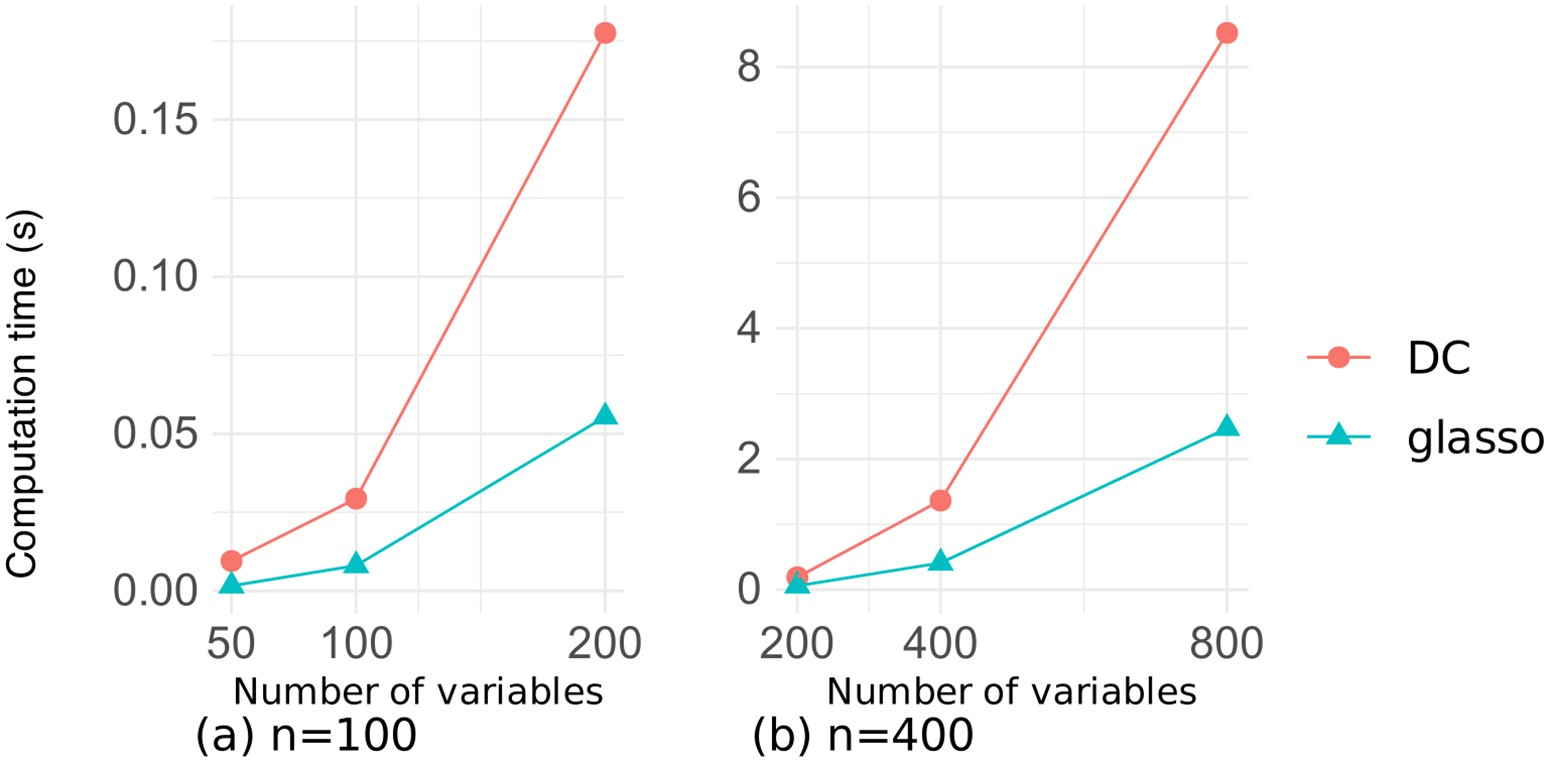
Computation time as a function of the number of variables on the chain graph dataset.

Figs 11 and 12 illustrate the relationship between the number of variables and the computation time for estimation on the datasets of random and chain graphs, respectively, with sample sizes *n* ∈ {100, 400}. There was a little difference in the computation time between the two datasets, and our DC method took about four times longer than did the glasso method. This is due to the two reasons, namely the repeated execution of the graphical lasso algorithm, and the repeated eigenvalue calculations in tuning the penalty parameter *η* in Algorithm 2. However, both methods took less than 1.5 seconds for *p* ≤ 400, and our DC method converged in approximately 8 seconds even for *p* = 800, demonstrating that our algorithm was sufficiently fast.

**Fig 13:**
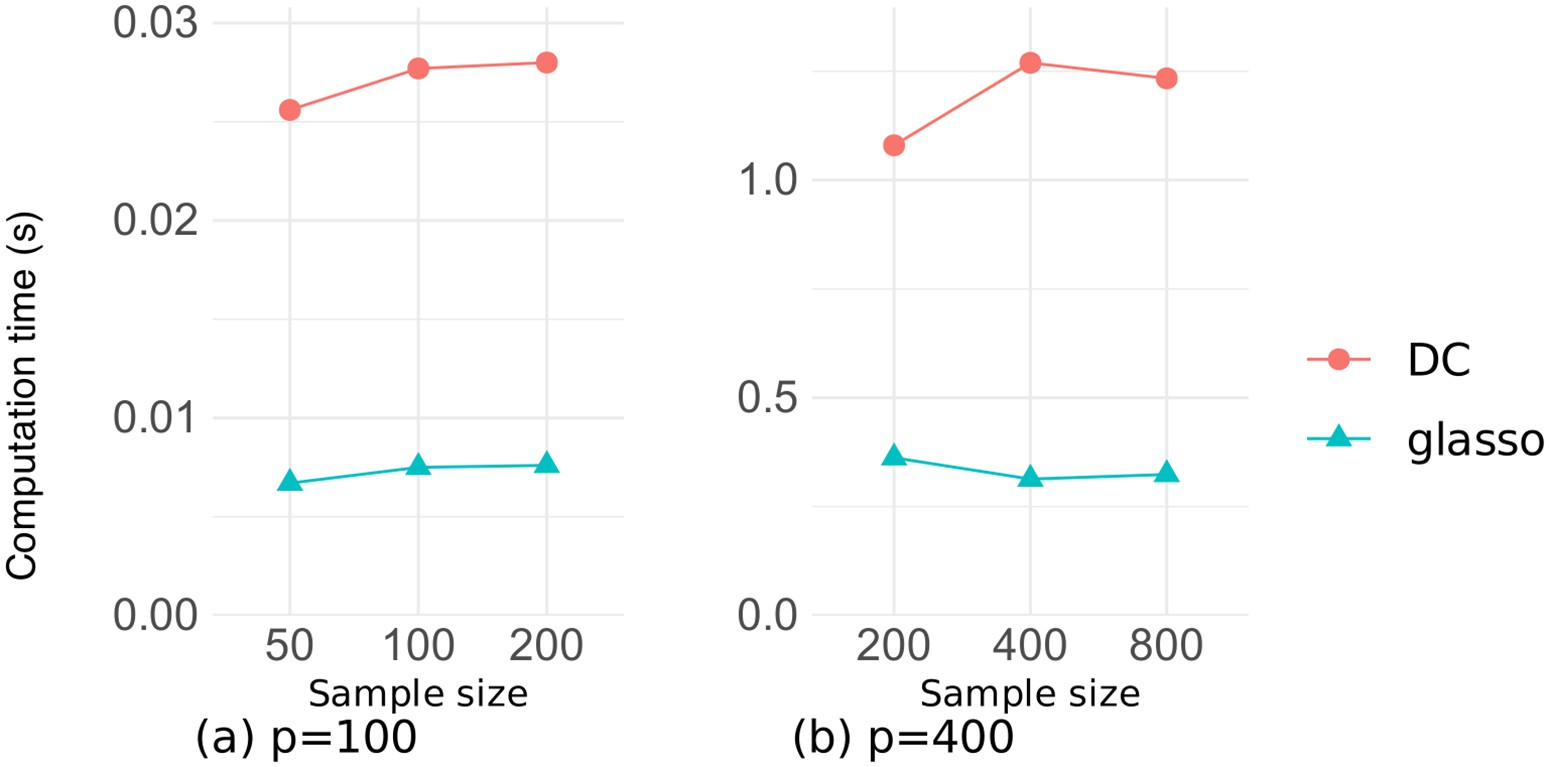
Computation time as a function of the sample size on the random graph dataset.

**Fig 14:**
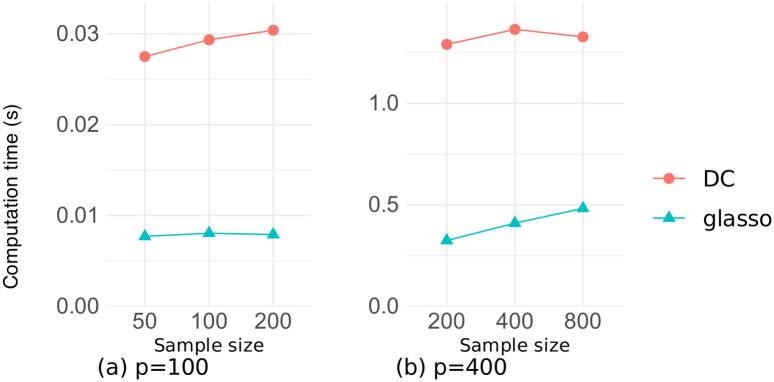
Computation time as a function of the sample size on the chain graph dataset.

Figs 13 and 14 illustrate the relationship between the sample size and the computation time for estimation on the datasets of random and chain graphs, respectively, where the number of variables is *p* ∈ {100, 400}. These figures confirm that the computation time for both methods was strongly dependent on the number of variables and changed very little even when the sample size was increased several times.

Table 1 lists the average numbers of iterations and eigenvalue calculations required by our DC algorithm. Recall here that the DC algorithm executes the graphical lasso algorithm at each iteration and repeatedly calculates the eigenvalues to tune the penalty parameter *η*. We can see from Table 1 that the DC algorithm terminated in only two iterations and calculated the eigenvalues around ten times.

**Table 1:** Numbers of iterations (#Ite) and eigenvalue calculations (#Eig) in the DC algorithm on the random and chain graph datasets.

*p*	*n*	Random		Chain	
		#Ite	#Eig	#Ite	#Eig
100	50	2.0	8.0	2.0	8.0
	100	2.0	8.0	2.0	10.0
	200	2.0	10.0	2.0	10.0
400	200	2.0	10.0	2.0	10.0
	400	2.0	10.0	2.0	12.0
	800	2.0	10.0	2.0	12.0

## Conclusion

We considered estimation of sparse Gaussian graphical models using the cardinality constraint based on the *ℓ*_0_ norm. We reformulated the sparse estimation problem with the cardinality constraint as an unconstrained penalty form using the largest-*K* norm. To solve this problem efficiently, we designed a DC algorithm that repeatedly executes the graphical lasso algorithm.

To verify the performance of our method, we conducted computational experiments using two types of synthetic datasets. In the experiments where the number of edges was selected through cross-validation, our method estimated conditional independence graphs more accurately than did other conventional methods. In the experiments where the number of selected edges was given, our method outperformed the graphical lasso and SCAD regularization and was comparable to the adaptive lasso in terms of the edge selection accuracy. In addition, our method took only about four times as long as the graphical lasso, indicating that the computation of our algorithm is fast enough for practical use.

A future direction of study will be to overcome computational challenges of our algorithm for sparse GGM estimation. As for the computational efficiency, Nakayama and Gotoh [42] reported that proximal gradient methods outperformed DC algorithms in some aspects of sparse regression, and Zhou et al. [43] proposed a proximal alternating direction method of multipliers for DC optimization problems. Additionally, since our method solves a penalized form of the problem, the obtained solutions do not always satisfy the original cardinality constraint. Another direction of future research will be to extend our method to multivariate time series analysis [44–46].
